# Supplementary material for: δ-Catenin controls astrocyte morphogenesis via layer-specific astrocyte–neuron cadherin interactions
Source: J Cell Biol. 2023 Sep 14;222(11):e202303138. doi: 10.1083/jcb.202303138 (PMC10501387; doi:10.1083/jcb.202303138)
Supplement: SourceData F3 — is the source file for Fig. 3. [file JCB_202303138_SourceDataF3.pdf]

Original image. Ladder used: Precision Plus Protein Kaleidoscope

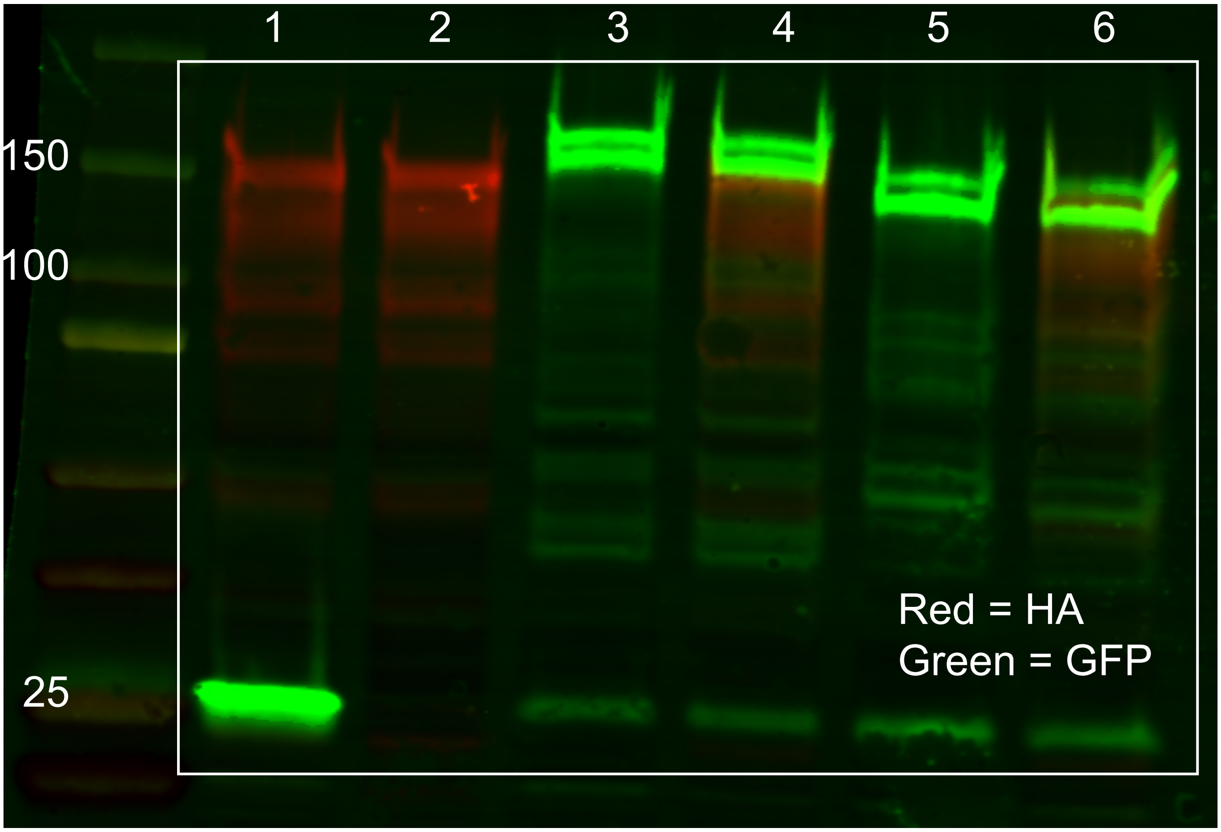

Input blot

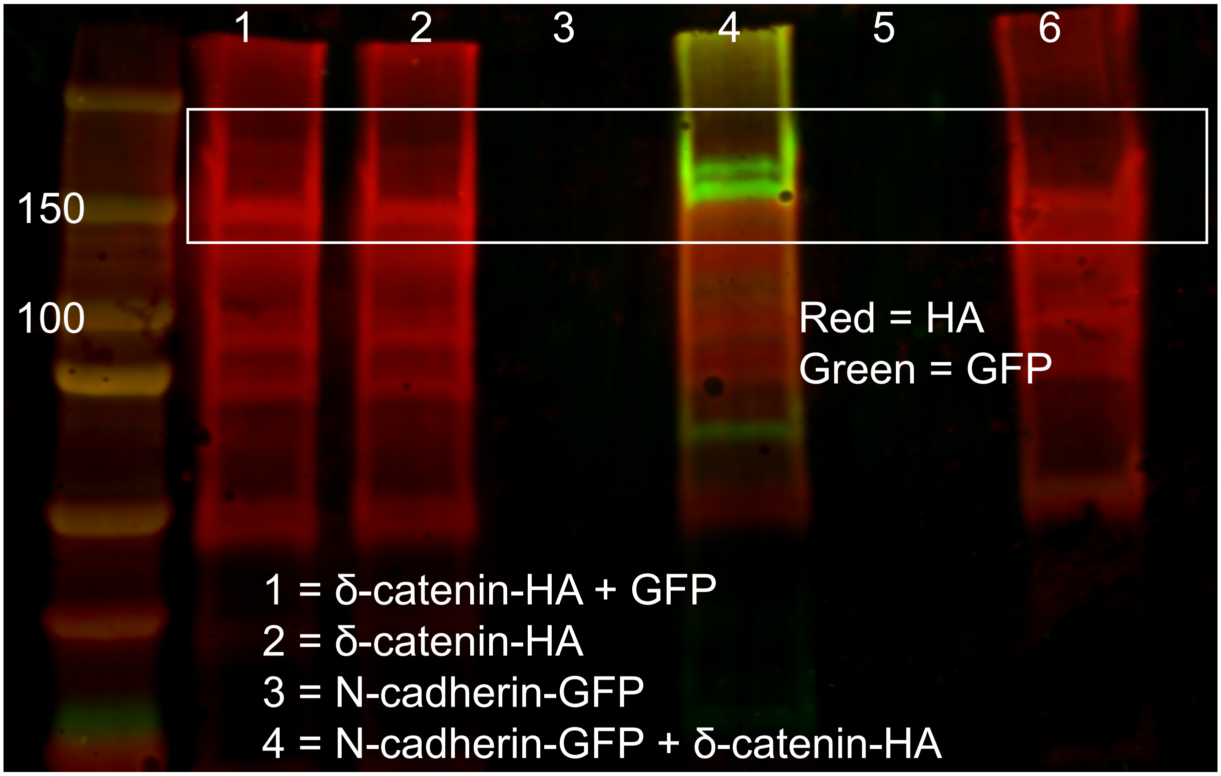

IP blot

1 = δ-catenin-HA + GFP
2 = δ-catenin-HA
3 = N-cadherin-GFP
4 = N-cadherin-GFP + δ-catenin-HA
5 = N-cadherinΔJMD-GFP
6 = N-cadherinΔJMD-GFP + δ-catenin-HA

Original image. Ladder used: Precision Plus Protein Kaleidoscope

Input blot 1

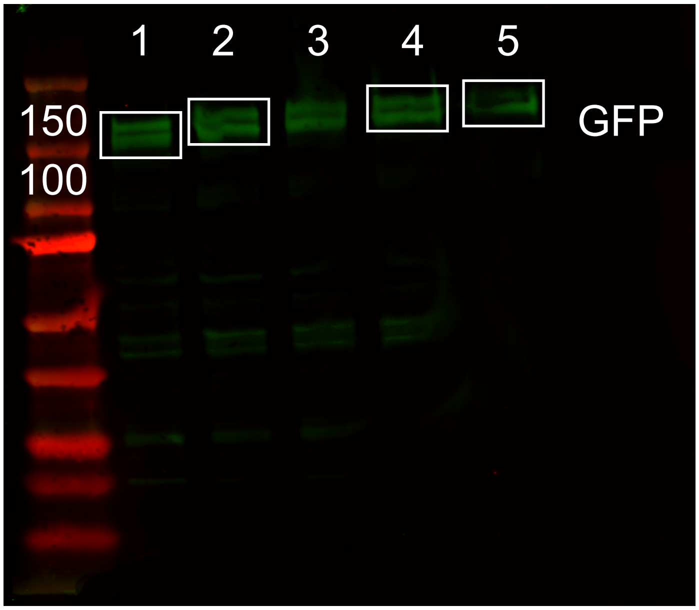

Input blot 2

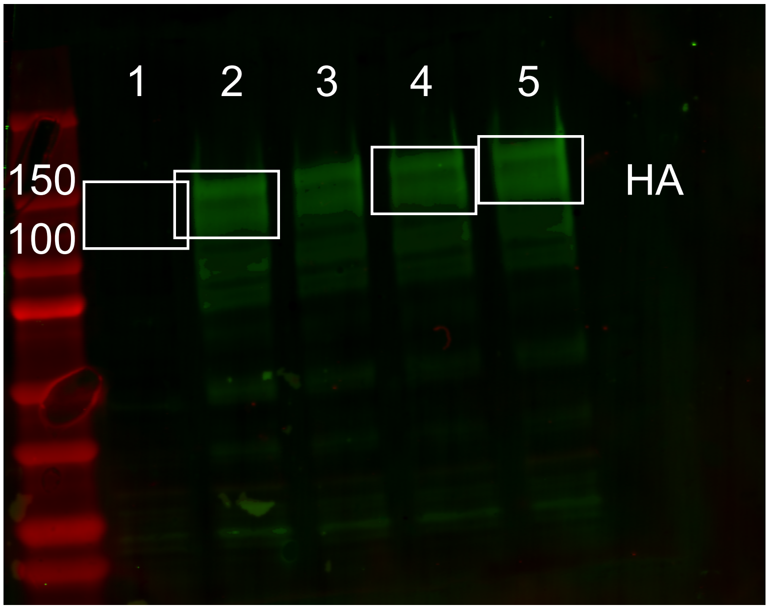

IP blot 1

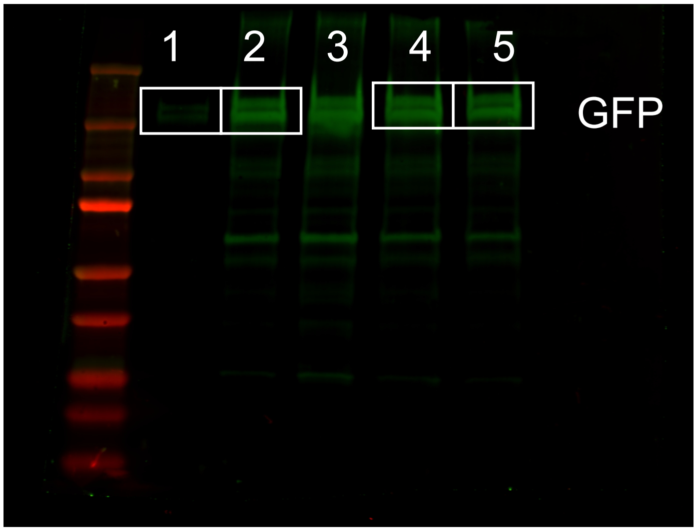

IP blot 2

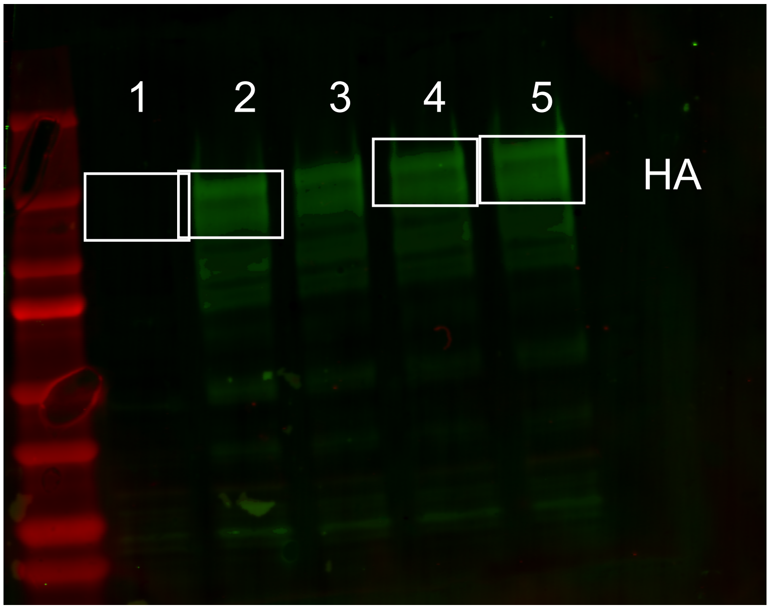

1 = N-cadherin-GFP
2 = N-cadherin-GFP + δ-catenin-HA
3 = N-cadherin-GFP + δ-catenin-R720A-HA (not discussed in manuscript)
4 = N-cadherin-GFP + δ-catenin-R713C-HA
5 = N-cadherin-GFP + δ-catenin-G810R-HA

Original image. Ladder used: Precision Plus Protein Kaleidoscope

Input blot

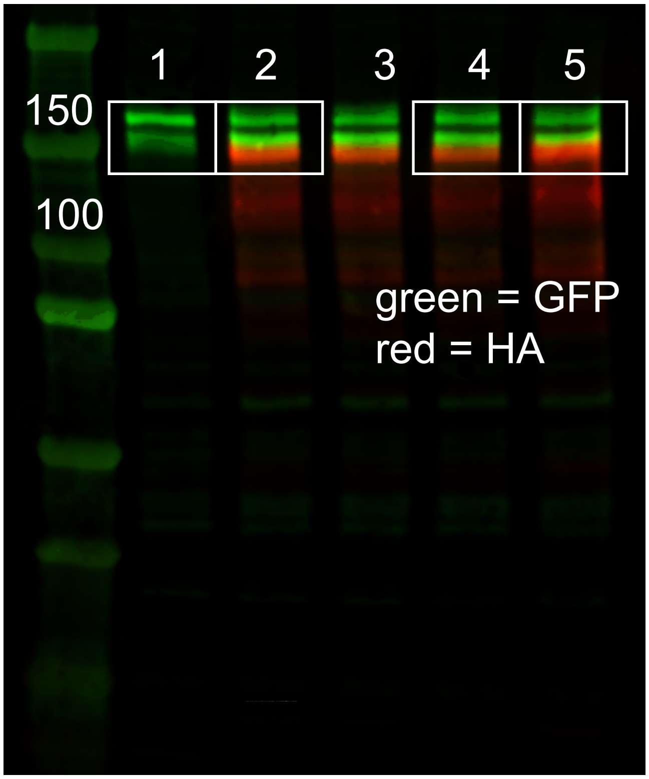

No-stain protein labeling of input blot

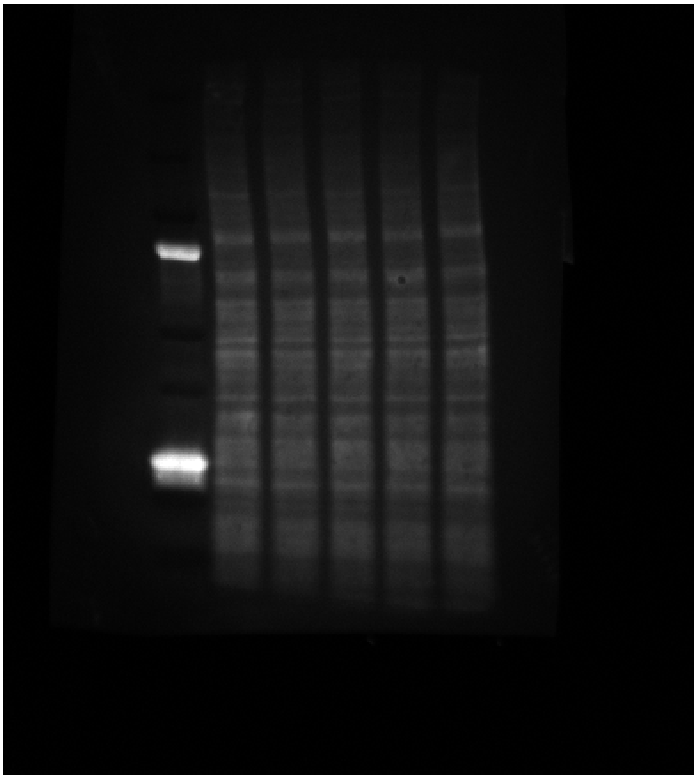

IP blot

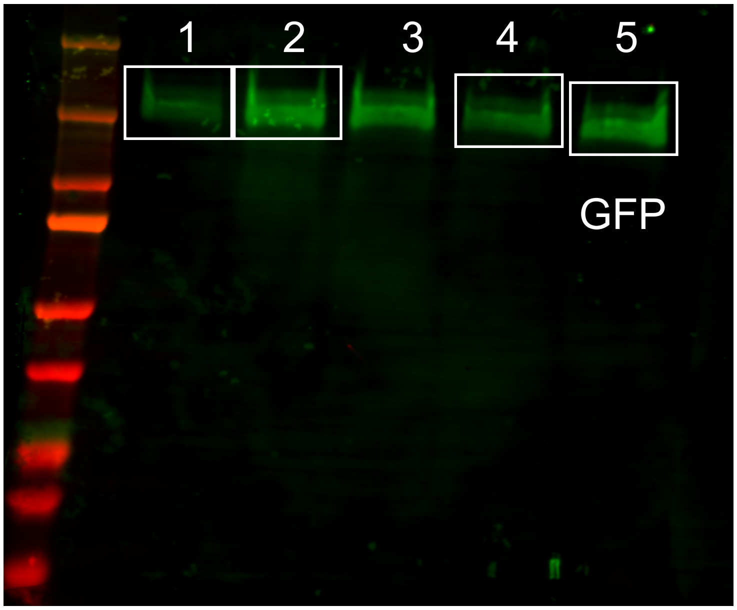

IP blot quantified on Odyssey Clx imager

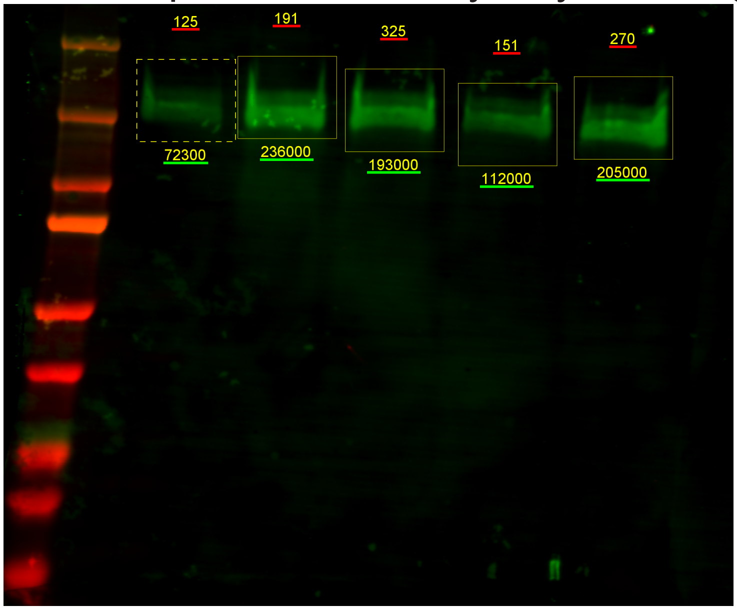

1 = N-cadherin-GFP
2 = N-cadherin-GFP + δ-catenin-HA
3 = N-cadherin-GFP + δ-catenin-R720A-HA (not discussed in manuscript)
4 = N-cadherin-GFP + δ-catenin-R713C-HA
5 = N-cadherin-GFP + δ-catenin-G810R-HA
