# Supplementary material for: δ-Catenin controls astrocyte morphogenesis via layer-specific astrocyte–neuron cadherin interactions
Source: J Cell Biol. 2023 Sep 14;222(11):e202303138. doi: 10.1083/jcb.202303138 (PMC10501387; doi:10.1083/jcb.202303138)

Original image. Ladder used: Precision Plus Protein Kaleidoscope

Input blot for δ-catenin

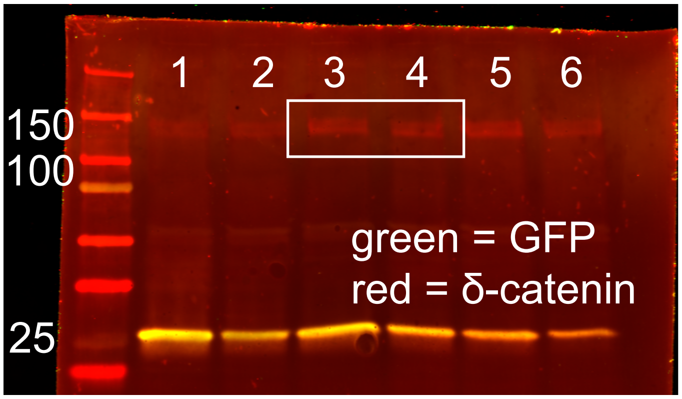

No-stain protein labeling of input blot

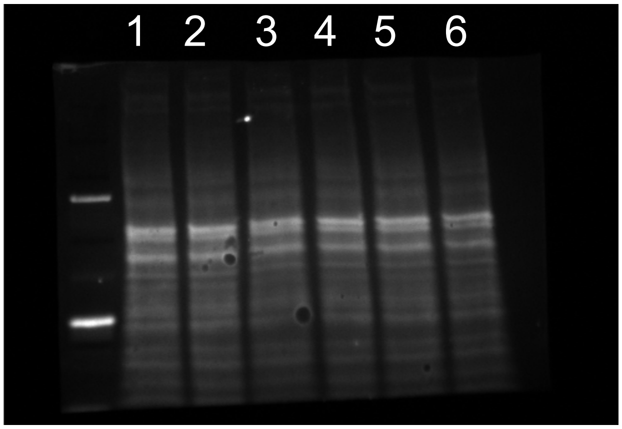

1 = shControl Set 1
2 = shCtnnd2 Set 1
3 = shControl Set 2
4 = shCtnnd2 Set 2
5 = shControl Set 3
6 = shCtnnd2 Set 3

Input blot for total N-cadherin

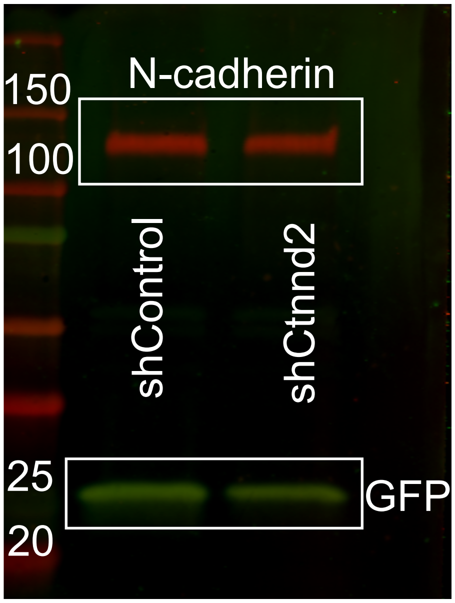

IP blot for surface N-cadherin

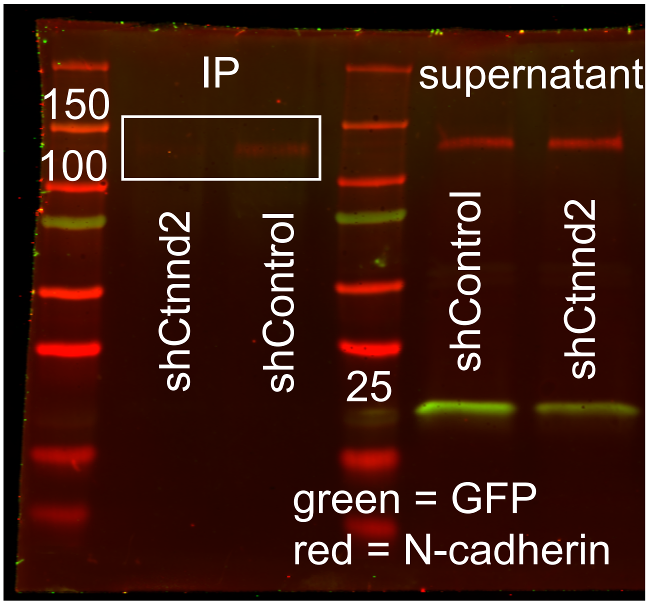

N-cadherin input and IP blots quantified on Odyssey Clx imager

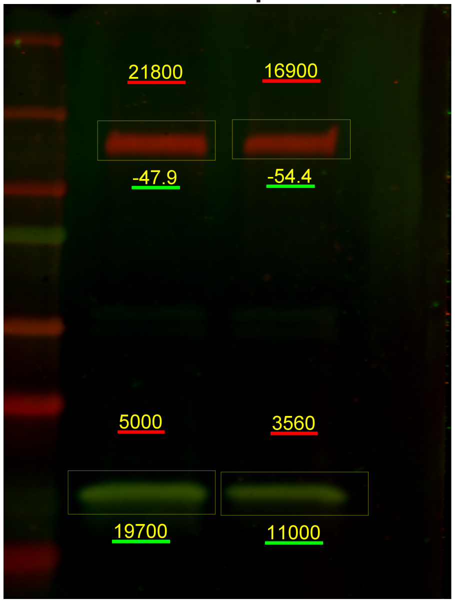

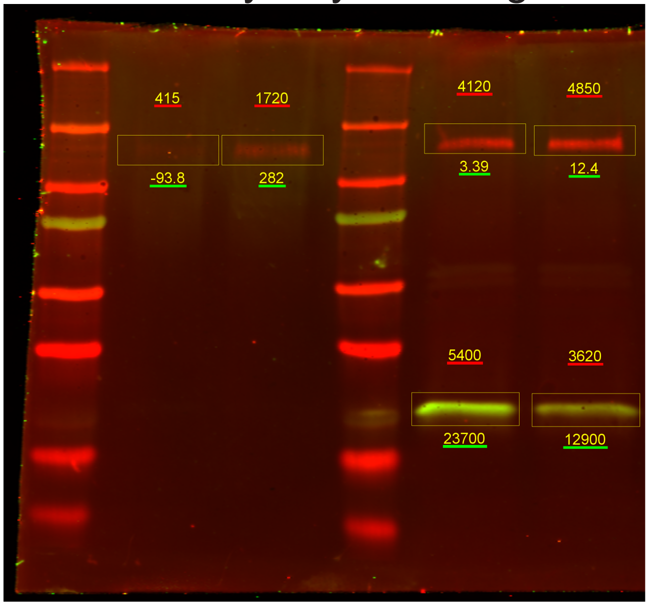

Supplement: SourceData F4 — is the source file for Fig. 4. [file JCB_202303138_SourceDataF4.pdf]
