# Supplementary material for: δ-Catenin controls astrocyte morphogenesis via layer-specific astrocyte–neuron cadherin interactions
Source: J Cell Biol. 2023 Sep 14;222(11):e202303138. doi: 10.1083/jcb.202303138 (PMC10501387; doi:10.1083/jcb.202303138)

Original image. Ladder used: Precision Plus Protein Kaleidoscope
Membrane was cut after blocking and before 1Ab incubation

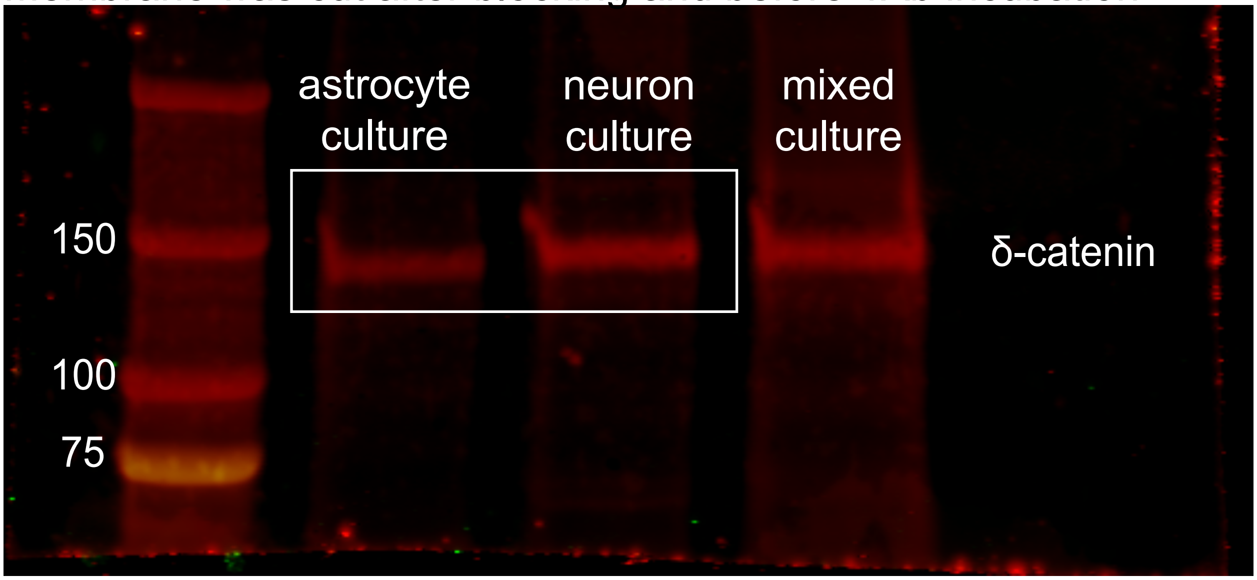

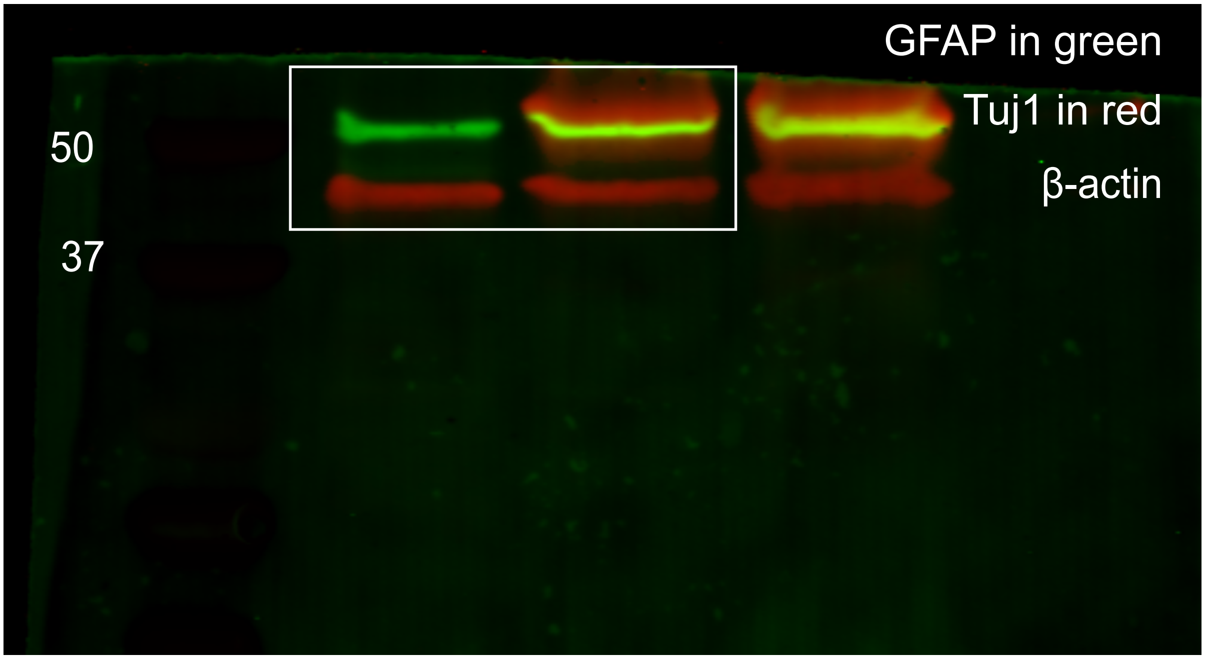

Supplement: SourceData FS1 — is the source file for Fig. S1. [file JCB_202303138_SourceDataFS1.pdf]
