# Supplementary material for: δ-Catenin controls astrocyte morphogenesis via layer-specific astrocyte–neuron cadherin interactions
Source: J Cell Biol. 2023 Sep 14;222(11):e202303138. doi: 10.1083/jcb.202303138 (PMC10501387; doi:10.1083/jcb.202303138)
Supplement: SourceData FS2 — is the source file for Fig. S2. [file JCB_202303138_SourceDataFS2.pdf]

Original image. Ladder used: Precision Plus Protein Kaleidoscope

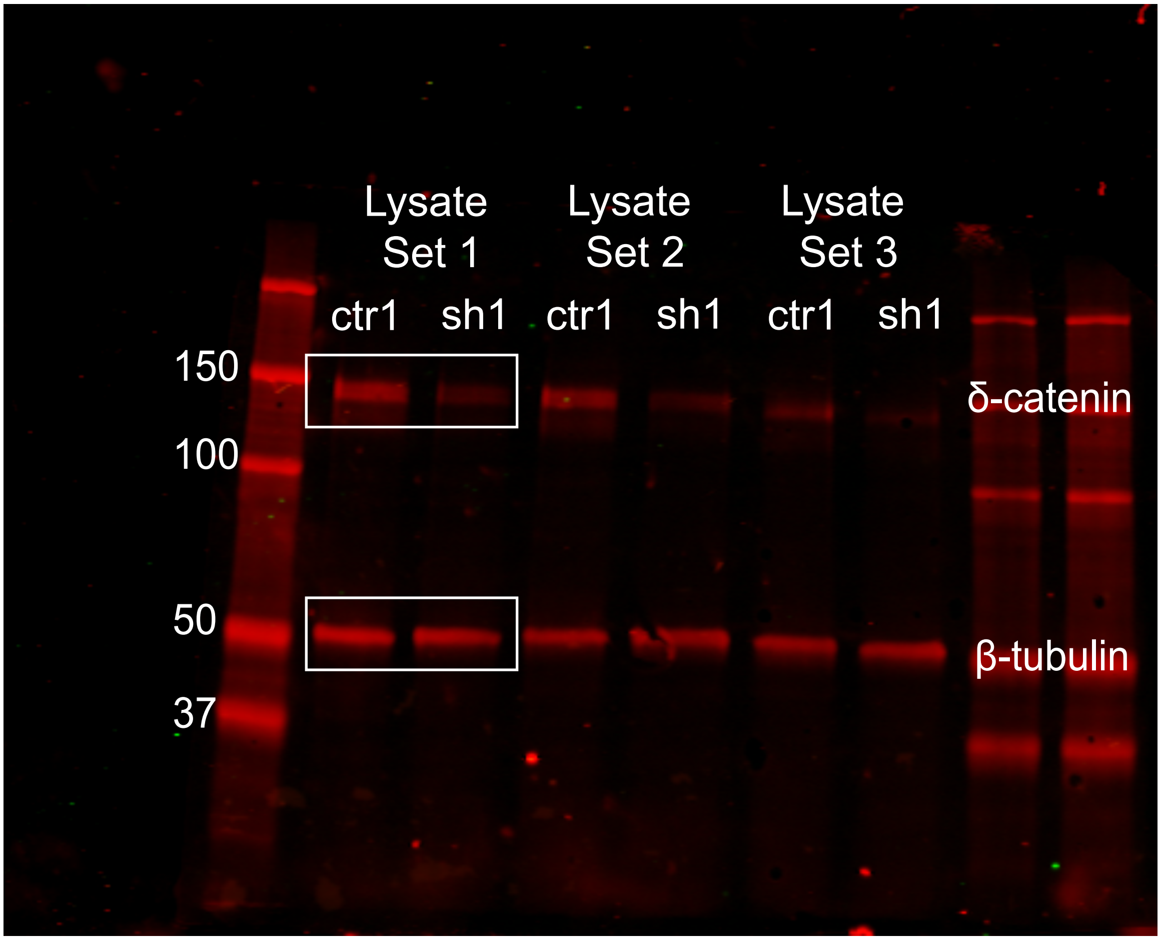

Imaged as quantified on Odyssey Clx Imager

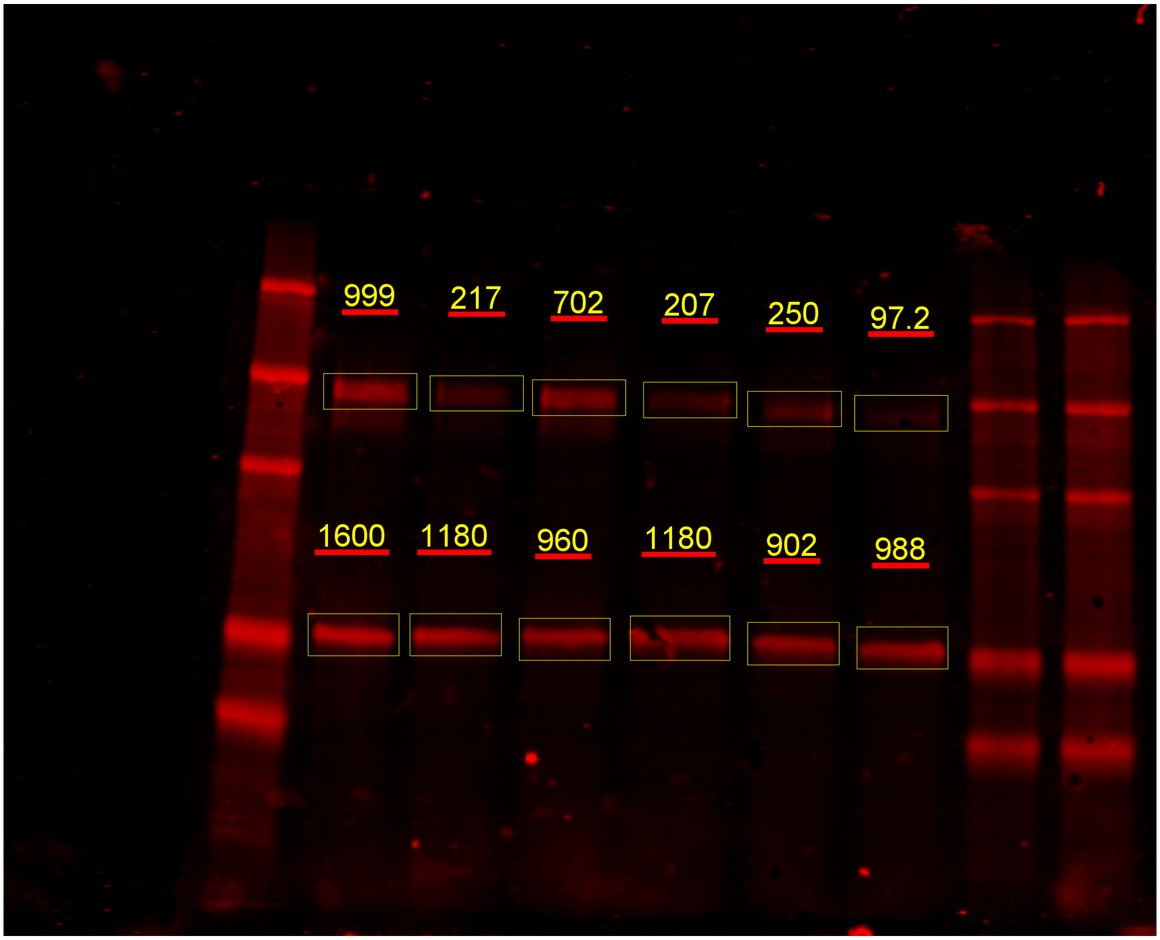

Original image. Ladder used: Precision Plus Protein Kaleidoscope

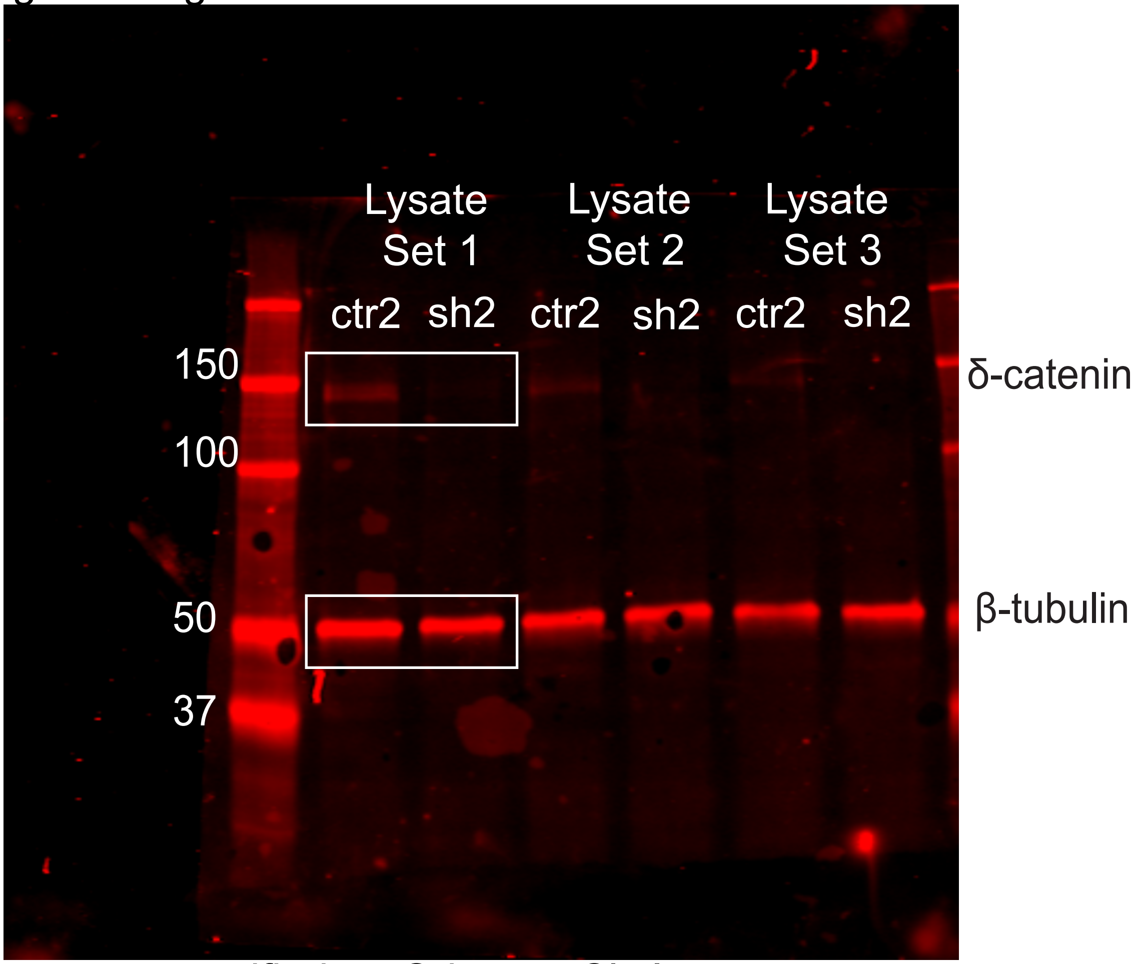

Image as quantified on Odyssey Clx Imager

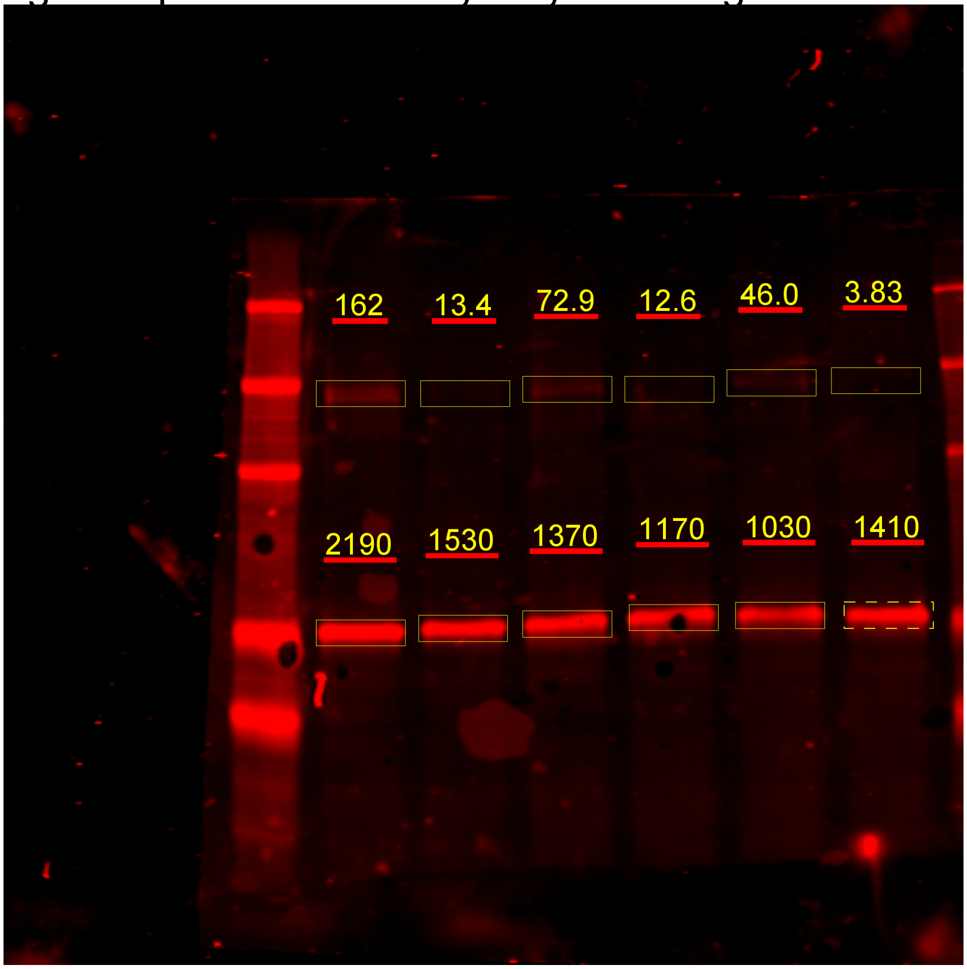

Original image. Ladder used: Precision Plus Protein Kaleidoscope

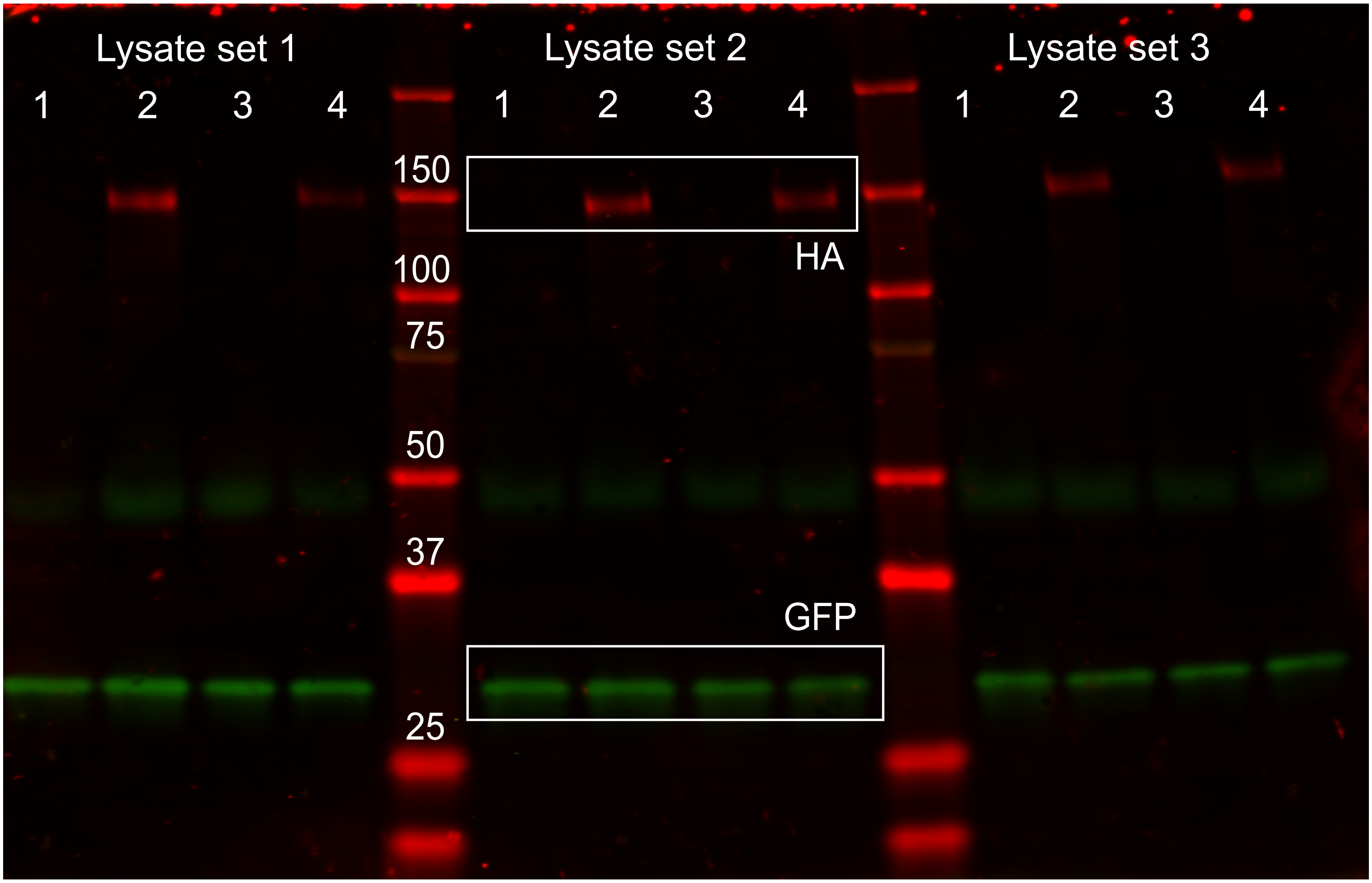

1 = shControl-GFP
2 = shControl-GFP + hCTNND2-HA
3 = shCtnnd2-GFP
4 = shCtnnd2-GFP + hCTNND2-HA
